# Pheromone modulates two phenotypically plastic traits – adult reproduction and larval diapause – in the nematode *Caenorhabditis elegans*

**DOI:** 10.1186/s12862-017-1033-9

**Published:** 2017-08-22

**Authors:** Barney Wharam, Laura Weldon, Mark Viney

**Affiliations:** School of Biological Sciences, University of Bristol, Tyndall Avenue, Bristol, BS8 1TQ UK

**Keywords:** *C. elegans*, Pheromone, Reproduction, Dauer, Ascaroside

## Abstract

**Background:**

Animals use information from their environment to make decisions, ultimately to maximize their fitness. The nematode *C. elegans* has a pheromone signalling system, which hitherto has principally been thought to be used by worms in deciding whether or not to arrest their development as larvae. Recent studies have suggested that this pheromone can have other roles in the *C. elegans* life cycle.

**Results:**

Here we demonstrate a new role for the *C. elegans* pheromone, showing that it accelerates hermaphrodites’ reproductive rate, a phenomenon which we call pheromone-dependent reproductive plasticity (PDRP). We also find that pheromone accelerates larval growth rates, but this depends on a live bacterial food source, while PDRP does not. Different *C. elegans* strains all show PDRP, though the magnitude of these effects differ among the strains, which is analogous to the diversity of arrested larval phenotypes that this pheromone also induces. Using a selection experiment we also show that selection for PDRP or for larval arrest affects both the target and the non-target trait, suggesting that there is cross-talk between these two pheromone-dependent traits.

**Conclusions:**

Together, these results show that *C. elegans*’ pheromone is a signal that acts at two key life cycle points, controlling alternative larval fates and affecting adult hermaphrodites’ reproduction. More broadly, these results suggest that to properly understand and interpret the biology of pheromone signalling in *C. elegans* and other nematodes, the life-history biology of these organisms in their natural environment needs to be considered.

## Background

In nature, animals compete for limiting resources, with the ultimate aim of maximizing their reproductive success, and so their individual evolutionary fitness. In this quest, animals use information from their environment to make decisions, including how best to find and exploit limiting resources. These decisions are shaped, moulded and optimized by natural selection, to contribute to individuals’ evolutionary fitness.

There are many examples of the types of decisions that animals make. One set of examples are animals’ behavioural repertories that they use, for example to locate food or mates. Animals also make phenotypically plastic developmental decisions, for example where a juvenile form chooses between alternative adult fates. One striking example of this are some species of the water flea *Daphnia* spp., where developing juveniles can choose between growing into one of two adult morphs: (i) a helmeted form, which has a protective anterior spike, with development of this form being induced in the presence of potential predators, (ii) or a helmet-less form, which occurs in the absence of predators [1]. Here, developing animals use signals from their environment (presence or absence of predators) to make a decision about which adult morph to grow into. In this scenario, it is presumed that there is a cost to growing the protective spike, such that doing so is only worthwhile in the likely presence of a predator.

The free-living nematode *Caenorhabditis elegans* is a widely used model system. Its life cycle contains a phenotypically plastic decision. Indeed among both free-living and parasitic nematodes more widely there are many species with such decisions [2, 3]. For *C. elegans*, juvenile worms at the second larval stage (L2) can either (i) continue to grow and develop (via an L3 and L4 stage) into reproductive adults, (ii) or they can arrest their development by moulting into a specialised L3 stage, called a dauer larva [4]. Dauer larvae are long lived and environmentally resistant, and persist in the environment until at some later point when conditions improve, they resume their development by moulting to the L4 stage, and then into reproductive adults.

Juvenile worms make the decision between growth into reproductive adults or arrested larval development based on their sensation of both a *C. elegans* pheromone (which is produced by all worms) and of food [4]. The concentration of pheromone in the environment is thought to be a measure of con-specific population density (though see below). The development of the arrested dauer larval form is usually favoured when there is a high concentration of pheromone and a low concentration of food, and high temperature, together potentially signalling many other *C. elegans* worms with rather little food among them. Alternatively, growth to reproductive adulthood is favoured in the converse conditions, potentially signalling abundant food and few other *C. elegans* worms.

In the wild, *C. elegans* lives in rotting vegetation, which is a boom-and-bust environment where occasional periods of high food abundance are separated by periods when food is scarce [5]. In these environments the arrested dauer larva form is an adaptation to survive when food is absent or scarce. Indeed, dauer larvae are the life cycle stage most commonly found in the wild [6–8], likely testifying to the common lack of available food in *C. elegans*’ environment.

The *C. elegans* dauer larva-inducing pheromone consists of a complex mixture of ascarosides and related molecules [9–11]. The ascaroside mixture produced by *C. elegans* varies across different life cycle stages [12], between sexes [13], and is affected by the worms’ diet [12], and nutritional status [9, 14]. Worms also appear to alter their ascaroside production in response to sensory information, for example worm density [13, 15, 16]. Detailed analysis of the biosynthesis of *C. elegans* ascarosides has allowed investigation of the biological activity of individual synthetic ascarosides [9, 10]. The results of these bioassays are consistent with the *C. elegans* pheromone being a modular library of signalling molecules [9, 10]. Very recently, effects of population density on worms’ developmental rate have been shown to be due to components of pheromone that are not ascarosides or other known steroid hormones, pointing to the continuing discovery of the bio-active components of *C. elegans* pheromone [16]. These range of different studies also point to the different approaches that can be used in understanding the effect of pheromone on worms [9–16]. One approach is to perform chemical assays on the excretomes of populations; a second approach is to sample and phenotype worms from growing populations of fixed population densities [16], whereas a third approach is to assay the effect of pheromone, or component molecules, on specific traits of interest – this is the approach we have used here. A diversity of approaches to studying pheromone is likely to be needed to fully elucidate the multiple effects of *C. elegans* pheromone*.*

The pheromone produced by different *C. elegans* strains has been compared, showing that it differs among strains, measured both as the dauer larva formation phenotype [17], and by the behavioural effects that it can induce [18]. Also, when different *C. elegans* strains are tested against standard, chemically synthesised ascaroside molecules, then there are strain-specific dauer larva formation and behavioural phenotypic responses too [17, 18]. Together, this means that different *C. elegans* strains actually both produce their own type of pheromone and that they have their own specific response to a pheromone or ascaroside signal [17, 18], with these different behavioural effects being at least in part due to genetic variation in genes coding for chemoreceptor molecules [19]. More broadly, this among-strain diversity of pheromone production, and response to pheromone, is also seen in the nematode *Pristionchus pacificus* [20], suggesting that such phenomena may be widespread among nematodes.

The highly dynamic composition of *C. elegans* pheromone – being dependent on worms’ sex, strain, and state – also suggests that the pheromone is not necessarily a straightforward, honest, species-wide signal of *C. elegans* population density. Instead, an alternative hypothesis is that the pheromone is a complex signal, potentially providing information about the strain composition and physiological state of neighbouring worms. Under this scenario, the pheromone may be being used by developing worms to make the phenotypically plastic decision between growth into reproductive adults, or into arrested larvae, but where this decision is based on an information-rich signal about the number and state of competing worms, possibly also including information about the presence of kin and of non-kin [17]. Also, following from this is the possibility that the pheromone signal is also used by other life cycle stages, including reproductive adults. Indeed, there are data supporting this; for example, *C. elegans* male-derived pheromone has been shown to accelerate hermaphrodites’ development and the maintenance of their germline precursor cells [16]; *C. elegans* pheromone has also been shown to increase adult worm lifespan and fecundity [21], as well as having multiple behavioural effects on worms [11, 18]. The biology of reproduction of adult worms (for example, when to start reproduction, and the relative number and quality of offspring) are key components of reproductive success, and if these traits are, at least in part, modulated by pheromone signals, then this is a further way in which *C. elegans* pheromone signalling can contribute to fitness.

With this perspective of the potential multiple roles of pheromone in nematode biology, here we have investigated how the *C. elegans* pheromone affects adult hermaphrodites’ reproduction as well as larval growth rates, finding that the pheromone speeds larval growth and hermaphrodite reproduction. This new role for *C. elegans* pheromone means that in its life cycle the pheromone acts on two key stages: (i) larvae that are deciding whether or not to arrest their development and (ii) on those that decide not to arrest, but instead to grow into adults and reproduce. In light of this dual role of pheromone, we also investigated how selection on each of these plastic traits (dauer vs. non-dauer larval development, and the timing of adult reproduction) affected the phenotypic response to both plastic traits, finding evidence of cross-talk between these traits. These multiple effects of *C. elegans* pheromone emphasise that a new interpretation of the role of this pheromone in the *C. elegans* life cycle is needed.

## Results

### Pheromone speeds egg laying

**Fig. 1:**
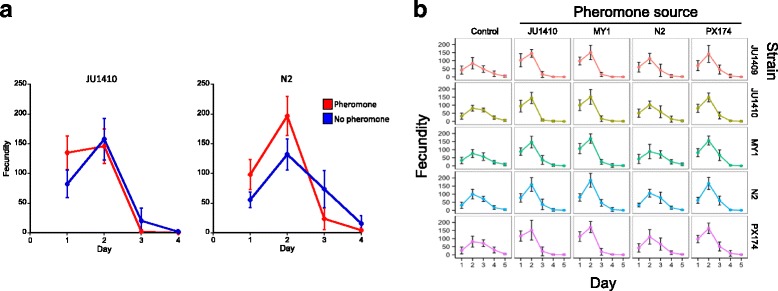
**a** The daily fecundity of JU1410 and N2 with or without pheromone derived from their own strain. Daily fecundity, as a proportion of lifetime fecundity, significantly varies across the four days of reproduction in both strains (JU1410 Day d.f. = 5, χ^2^ = 11,475, *P* < 0.001; N2 Day d.f. = 6, 11,445, *P* < 0.001; for JU1401 there are no day 4 data hence the difference in the relevant degrees of freedom) and there is a significant Treatment x Day interaction (JU1410 d.f. = 5, χ^2^ = 603.9, *P* < 0.001; N2 d.f. = 6, χ^2^ = 11,445, *P* < 0.001), showing that pheromone affects the timing of reproduction. **b** The daily fecundity of JU1409, JU1410, MY1, N2 and PX174 with or without pheromone derived from JU1410, MY1, N2, PX174, or in the control, no pheromone, treatment. Daily fecundity, as a proportion of lifetime fecundity, is changed by the presence of pheromone (Treatment x Day d.f. = 46, χ^2^ = 12,998, *P* < 0.001), and the strains differ in their responses (Strain x Treatment d.f. = 62, χ^2^ = 84, *P* = 0.001; Day x Strain x Treatment, d.f. = 24,132.5, χ^2^ = 1155.2 *P* < 0.001). Error bars are ±1 standard deviation

*C. elegans* lays eggs in the first four days of adulthood and we asked how this reproduction was affected by the presence of pheromone. We did this by comparing worms’ daily viable egg production in the presence or absence of pheromone. We found that pheromone accelerates egg laying, so that a greater proportion of worms’ lifetime reproduction occurs earlier when pheromone is present (Fig. 1a). For example, strain JU1410’s day 1 reproduction, as a proportion of lifetime reproduction, increases from 31 to 47% in the presence of pheromone; for strain N2 it increases from 20 to 30%. We call this phenomenon of acceleration of reproduction, pheromone-dependent reproductive plasticity (PDRP).

### *C. elegans* strains differ in their PDRP

**Fig. 2:**
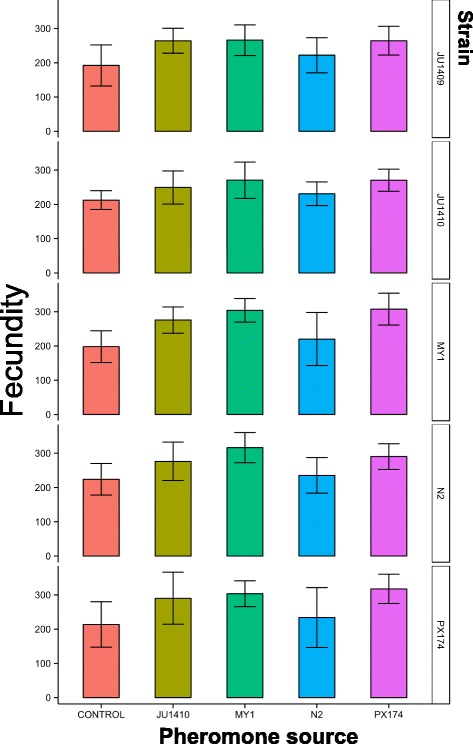
The lifetime fecundity of strains JU1409, JU1410, MY1, N2 and PX174 (right hand labels) in the presence of pheromone derived from JU1410, MY1, N2 and PX174, or the no pheromone control (bottom labels). Error bars are ±1 standard deviation. Lifetime fecundity differed among the different pheromone and control treatments (d.f. = 4, χ^2^ = 146.4, *P* < 0.001) with pheromone from JU1410, MY1, PX174 and N2 significantly increasing (*P* < 0.001; *P* < 0.001; *P* < 0.001; *P* = 0.046, respectively) fecundity compared with the control treatment. Post hoc Tukey analyses showed that pheromone derived from MY1, JU1410 and PX174 are not significantly different from one another in their effect on lifetime fecundity (*P* = 0.08, *P* = 0.07, *P* = 1.00, respectively), whereas that of N2 is different compared to MY1, JU1410 and PX174 (all *P* < 0.001). There was no significant difference in the way that each strain responded to the treatments (Strain x Treatment d.f. = 27, χ^2^ = 15.21, *P* = 0.51)

It has previously been shown that the chemical composition of pheromone differs among different *C. elegans* strains, and that different strains respond differently (measured as the dauer larva arrest phenotype) to the same pheromone mixture or purified ascaroside molecules [17]. Together, this means that there are a very wide range of among-strain pheromone interactions acting on this life-history trait. We therefore surveyed the PDRP trait among five *C. elegans* strains, tested with four different pheromone sources. This showed that PDRP was universal among these 20 strain-pheromone combinations (Fig. 1b). While PDRP shows the timing of strains’ reproduction, we also observed a pheromone-dependent increase in the trait of lifetime fecundity (average increase = 30%, range 5–55%) of the worms in these conditions (Fig. 2). However, the magnitude of the PDRP effect differed among the strains and pheromone source, meaning that different pheromone sources induced different levels of PDRP, and strains differed in the degree of PDRP when tested against the same source of pheromone (Fig. 1b). For example, strain MY1’s pheromone induced the greatest PDRP among the 5 strains, while N2’s pheromone was least effective at inducing PDRP. This finding is analogous to the pheromone effects on dauer larva formation, such that different *C. elegans* strains are qualitatively similar in forming dauer larvae after exposure to pheromone, but that they differ quantitatively in this trait [17].

### Pheromone accelerates *C. elegans*’ growth, but only if the food source is live

**Fig. 3:**
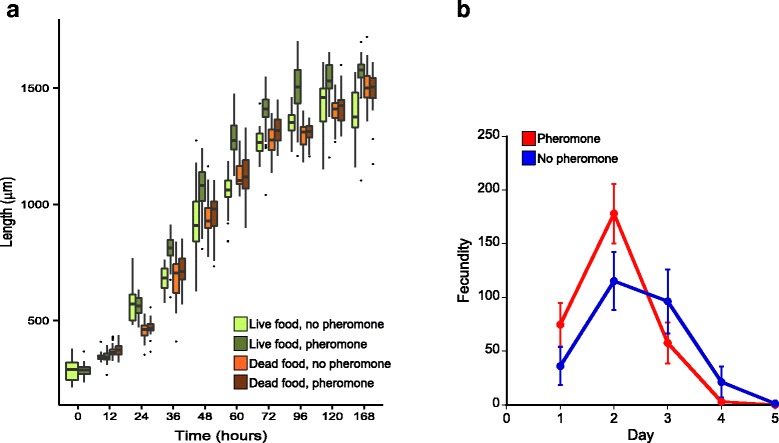
**a** N2 larvae grow faster in the presence of JU1410-derived pheromone when the bacterial food source is live so that at 36 h worms are bigger in the presence of pheromone (t(50.5) = −7.864, *P* < 0.001), and this difference continues into adulthood (Table 1). The growth-promoting effect of pheromone depends on the bacterial food source being alive, since with a dead food source pheromone treatment does not affect worm size at 36 h (t(78.6) = −1.5712, *P* = 0.12), or at any other times, except for 72 h (t(80) = −3.03, *P* = 0.003; Table 1). The boxplot shows the median worm length (horizontal line) and the box shows the inter-quartile range, with the lower whisker showing the lower quartile minus 1.5 times the interquartile range, and the upper whisker the upper quartile plus 1.5 times the interquartile range, with outliers shown as points. **b** The daily fecundity of N2 (± 1 standard deviation) in the presence or absence of JU1410-derived pheromone with a dead bacterial food source. Reproduction varies across the four days of reproduction (Day d.f. = 5, χ^2^ = 11,534, *P* < 0.001) and there is a significant Treatment x Day interaction (d.f. = 10, χ^2^ = 1795, *P* < 0.001), showing that PDRP occurs

We next asked how pheromone might be causing PDRP. We reasoned that since reproduction occurs as soon as larvae moult into adults [22], acceleration of this growth to adulthood could cause PDRP. We therefore measured the growth rate of *C. elegans* larvae in the presence or absence of pheromone. We found that larvae grew comparatively faster in the presence of pheromone, with this seen in two ways (Fig. 3a). Firstly, that larvae grew more rapidly in the presence of pheromone, such that after 36 h pheromone-exposed worms were an average of 804 (± 66 SD) μm long, while control worms were 684 (± 51) μm (Table 1). Secondly, that worms were at a more advanced developmental stage, such that after 36 h 92% (95% binomial confidence intervals 76–98%) of pheromone treated worms were between the mid fourth larval stage (L4) and the adult stage, compared with only 44% (95% binomial confidence intervals 27–63%) of control worms (Table 1).

**Table 1:** The size of N2 worms with or without JU1410-derived pheromone with (A) live or (B) dead *E. coli* OP50, showing the mean length (SD = standard deviation), and t-test results comparing the pheromone-treated and control worms (significant effects are in bold), and (C) the larval stage of development after 36 h of growth as judged by the development of the vulva as in (A) showing the number and, in parentheses, the proportion at each stage

	Without pheromone		With pheromone		t-test		
Time (h)	Length (μm)	SD	Length (μm)	SD	d.f.	*t*	*P*
(A)							
0	284.89	42.87	287.63	26.70	nd	nd	nd
12	344.45	17.54	342.08	27.65	33	0.34	0.74
24	573.36	76.46	560.87	45.07	33.4	0.67	0.51
**36**	**683.58**	**51.07**	**804.24**	**65.86**	**50.5**	**−7.86**	**<0.001**
**48**	**931.95**	**143.34**	**1061.76**	**106.71**	**43.3**	**−3.93**	**<0.001**
**60**	**1050.69**	**77.81**	**1282.13**	**84.13**	**74.8**	**−12.54**	**<0.001**
**72**	**1269.93**	**50.59**	**1399.32**	**95.65**	**57.4**	**−7.28**	**<0.001**
**96**	**1351.72**	**50.76**	**1496.66**	**113.61**	**50.7**	**−7.03**	**<0.001**
**120**	**1426.73**	**144.42**	**1524.65**	**116.55**	**30.6**	**−2.16**	**0.04**
**168**	**1399.29**	**119.21**	**1538.65**	**146.62**	**23.2**	**−2.73**	**0.01**
(B)							
12	369.8678	24.67	370.37	25.53	67.4	−0.08	0.94
24	454.81	33.69	470.23	31.05	74.8	74.79	0.04
36	685.24	91.94	713.83	71.71	78.6	78.59	0.12
48	946.89	79.87	961.37	81.85	84.0	83.95	0.41
60	1126.54	66.14	1127.45	92.39	67.88	−0.05	0.96
**72**	**1273.73**	**70.68**	**1316.78**	**57.96**	**80**	**−3.03**	**0.003**
96	1298.74	49.27	1311.07	39.78	88.47	−1.36	0.18
120	1402.26	55.53	1412.51	67.68	93.07	−0.83	0.41
168	1506.47	72.66	1493.70	77.99	97.51	0.85	0.40
(C)							
Developmental stage		Without pheromone			With pheromone		
Early L3		1 (0.04)			0 (0)		
Mid L3		0 (0)			0 (0)		
Late L3		1 (0.04)			1 (0.04)		
L3/L4 moult		3 (0.12)			0 (0)		
Early L4		9 (0.36)			1 (0.04)		
Mid L4		5 (0.2)			6 (0.23)		
Late L4		4 (0.16)			14 (0.54)		
Late L4 to adult		2 (0.08)			4 (0.15)		
Total		25 (1)			26 (1)		

L is larval stage. nd = done

In these assays, the worms were feeding on a live *E. coli* food source, as is standard for *C. elegans*. Noting that live food has been shown to shorten *C. elegans’* lifespan [23], we asked whether the growth- and development-promoting effects of pheromone were affected by whether or not this *E. coli* food was alive. We did this by comparing the growth of *C. elegans* larvae (in the presence of pheromone) on live and on dead food. We found that the pheromone’s growth-promoting effects only occurred in the presence of live food; the effects were abolished if the food was dead (Fig. 3a; Table 1). We prepared dead food by using agar plates containing Kanamycin (together with exposing the bacteria to UV illumination), and worms were also assayed on these kanamycin-containing plates, and therefore we can not fully exclude the possibility that kanamycin may contribute directly to the phenomena we have observed. We next asked whether the PDRP effect was also affected by whether the food source was alive or dead. This showed that the PDRP effect does not require live food (Fig. 3b). Because the growth-promoting effects of pheromone are dependent on the food source being live, but that PDRP is not dependent on the food source being live, this shows that the PDRP effect is not solely due to the larval growth promoting effects of pheromone.

### Selection on PDRP affects dauer larva formation, and vice versa

Taken together, these results show that *C. elegans* pheromone can act on two different life cycle stages – larvae and adults – to effectively delay or hasten adult reproduction, respectively. The pheromone acting at these two life cycle stages may do so completely separately, or these effects may be linked. Mechanistically, the details of how the pheromone signal is sensed and transduced will affect the extent to which these are separate or inter-related effects. Ultimately, the nature of selection acting on the dauer arrest and adult reproduction schedule will also affect the extent of cross-talk, or not, between these pheromone-dependent effects on these life-history traits.

**Fig. 4:**
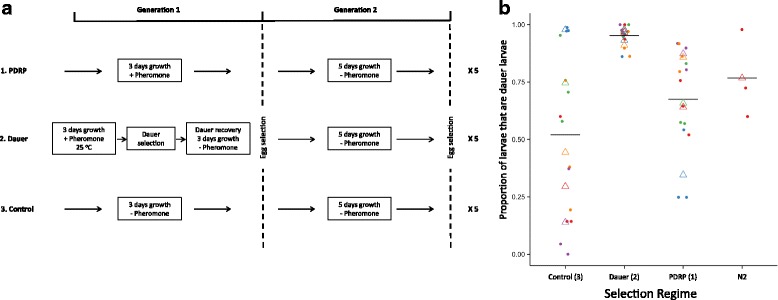
**a** Selection regimes 1 for PDRP, 2 for dauer larva formation, and 3 control, showing the first two generations of selection, which was repeated 5 times. **b** The proportion of dauer larva formed by lines selected for PDRP (selection regime 1), dauer larva formation (selection regime 2), or the control (selection regime 3), and strain N2, and selection regime significantly affected dauer larva formation (Selection Regime d.f. = 2 χ^2^ = 560, *P* < 0.001). For each line tested there were three replicates shown as dots, with their mean shown as triangles, with different colours showing different lines, and the overall mean for each selection environment is shown as a horizontal black line

To investigate the phenomenology of this, we conducted a selection experiment where worms were either selected for (1) PDRP, (2) dauer larva formation, or were (3) a control (Fig. 4a). In the PDRP regime 1, we alternately selected worms for fast reproduction in the presence of pheromone, and slow reproduction in its absence. In the dauer regime 2 we alternately selected for larvae to form dauer larvae before reproducing, and then for slow adult reproduction. In the control regime (3) we selected alternately for fast and slow reproduction, both in the absence of pheromone (Fig. 4a). After the lines had been selected, their dauer formation and PDRP phenotypes were determined, and we tested how selection for a target trait (e.g. PDRP) affected the target trait itself, as well as the non-target trait (e.g. dauer larva formation).

Considering the trait of dauer larva formation first, selection for (1) PDRP and (2) dauer larva formation, increased the dauer larva formation phenotype, compared with the (3) control selection regime. The (2) dauer selection regime had the strongest effect on the dauer larva formation trait (Fig. 4b). This result shows that selection for the target trait of dauer larva formation was successful, but that selection for the non-target trait also affected the target trait.

For the trait of PDRP, selection for (1) PDRP and (2) dauer larva formation both also resulted in a change in the PDRP phenotype, compared with the (3) control selection regime (Table 2A). While both regimes 1 and 2 affected PDRP, regime 1 had the strongest effect, shown by these worms’ greater reproduction on day 1 (Table 2B). Therefore, for the target trait of PDRP, both target selection and non-target selection affected the target trait.

**Table 2:** (A) GLMs describing worms’ daily fecundity, as a proportion of lifetime fecundity, with fixed effects including the Selection Regime ((1) PDRP, (2) Dauer, (3) Control) and the presence or absence of N2 pheromone (Treatment). The explanatory variables in the model, the d.f., the AIC, the χ^2^ value of the LRT and the associated *P* value are all shown. Models significantly different from the preceding model are indicated in bold. (B) A summary of post hoc contrasts between the three different selection regimes within each GLM describing the daily fecundity as a proportion of lifetime fecundity (as Table 2A), showing the *P* values, with significant values indicated in bold

(A)						
Models	Explanatory variables		d.f.	AIC	χ^2^	*P* value
day1_GLM1	Null		1	5540.52		
**day1_GLM2**	Treatment		**2**	**3882.30**	**1660.2**	**<0.001**
**day1_GLM3**	Treatment + Selection Regime		**4**	**3842.63**	**43.67**	**<0.001**
day2_GLM1	Null		1	3604.74		
**day2_GLM2**	Treatment		**2**	**3328.38**	**278.39**	**<0.001**
**day2_GLM3**	Treatment + Selection Regime		**4**	**3302.25**	**30.10**	**<0.001**
day3_GLM1	Null		1	7542.70		
**day3_GLM2**	Treatment		**2**	**4734.52**	**2810.2**	**<0.001**
**day3_GLM3**	Treatment + Selection Regime		**4**	**4710.01**	**28.52**	**<0.001**
day4_GLM1	Null		1	3878.1		
**day4_GLM2**	Treatment		**2**	**2618.27**	**1261.8**	**<0.001**
**day4_GLM3**	Treatment + Selection Regime		**4**	**2591.13**	**31.14**	**<0.001**
(B)						
Models						
		1. PDRP	2. Dauer			
day1_GLM3	**3. Control**	**<0.001**	**<0.001**			
day1_GLM3	**2. Dauer**	0.498	-			
day2_GLM3	**3. Control**	0.693	**<0.001**			
day2_GLM3	**2. Dauer**	**<0.001**	-			
day3_GLM3	**3. Control**	**<0.001**	0.94			
day3_GLM3	**2. Dauer**	**<0.001**	-			
day4_GLM3	**3. Control**	**<0.001**	**<0.001**			
day4_GLM3	**2. Dauer**	0.3805	-			

Together, these results show that selection for either PDRP or for dauer larva formation, was successful in selecting for these traits (compared with the control selection regime), but that in both cases these responses to selection also resulted in changes in the non-target trait. This therefore shows that these two pheromone-dependent, phenotypically plastic traits are not separate in the biology of *C. elegans.*

## Discussion

Animals use information from their environment to make decisions, ultimately to maximize their fitness. The nematode *C. elegans* has a pheromone signalling system where the pheromone signal differs among worms of different state, and where different worm strains also have different pheromone signals, and responses to them. Together, this therefore means that the *C. elegans* pheromone signalling system is potentially a very rich information resource that worms can use.

To date, the principal role of this pheromone has been thought to be the control of the induction of alternative larval fates (dauer vs. non-dauer). The results we present here show a clear second role for this pheromone, namely affecting the timing of adult worms’ reproduction. These results are therefore complementary to those showing how male-derived pheromone affects hermaphrodites’ germ line [23], and how pheromone can affect lifespan [21]. Interestingly, this previous work also showed that individual synthetic ascaroside molecules could also affect the magnitude of hermaphrodites’ fecundity [21]. These results therefore chime with ours, though it is important to emphasize that we show how pheromone can change the timing of hermaphrodites’ reproduction, rather than their total fecundity. It is noteworthy that a similar shift to early reproduction is observed when *C. elegans* is grown on some strains of pathogenic bacteria, although without the increase in total fecundity [24]. In this case, the shift to earlier reproduction, and its associated acceleration of ageing, has been suggested to be an adaptive response to high mortality rates [24]. Since high pheromone concentrations in the wild are thought to be associated with high population density, and so the exhaustion of food sources, also leading to high mortality rates, then the pheromone-dependent changes in reproduction and development described here may be an adaptive phenotype similar to the ageing modulation observed in worms grown on pathogenic bacteria. Very recent work shows how larval *C. elegans* density can accelerate hermaphrodites’ development [16] which is fully consistent with the results we present here. Interestingly, these effects appear to be due to hitherto unknown components of *C. elegans* pheromone [6], also consistent with our demonstration of these effects using whole pheromone. We also find that pheromone can accelerate worms’ growth rates, but that these effects require a live food source, whereas PDRP does not. We hypothesise that the growth-promoting effect of the pheromone is due to the bacteria metabolising components of the pheromone to produce products that then act as *C. elegans* growth factors. It is likely that the acceleration of growth caused by pheromone contributes, at least in part, to the changes in reproduction that we have observed, but further work will be required to determine if additional factors contribute to these reproductive changes.

We further show that the PDRP effect is qualitatively consistent across different *C. elegans* strains when exposed to pheromone from diverse sources, though we find that the PDRP effects differ quantitatively among strains. This is analogous to previous work showing how the dauer larva induction response to *C. elegans* pheromone (or synthesised ascaroside molecules) is broadly qualitatively consistent, but quantitatively different, among different strains [17]. More generally, these results, taken together, emphasize the importance of examining pheromone responses in a diversity of *C. elegans* strains, particularly noting that the standard lab strain N2 is often quite different phenotypically compared with more recently wild-isolated strains [17].

The results presented here, together with other work [16, 21, 23], now clearly show that *C. elegans* pheromone acts at least two points in the *C. elegans* life cycle: on larvae making a developmental choice, and on adult hermaphrodites’ reproduction. Deciding whether or not to undergo larval arrest and deciding the magnitude and timing of one’s reproduction are key life-history decisions that are likely to be major components of individuals’ fitness. These results also mean that a single pheromone signal needs to be able to provide the appropriate signal to each of these two decision points. Because of this we also asked how selection for pheromone responsiveness at each of these points would affect the target trait as well as the non-target trait. These results showed that selection for each trait did affect the target trait, as well as the non-target trait. This therefore implies that there is cross-talk among these two pheromone-dependent traits. The potential adaptive significance of this is not yet clear, since this cross-talk can be thought of as a constraint (such that one trait cannot evolve independently of the other) or as an inherent coordination between two phenotypic response to pheromone. Further work will be required to elucidate this. We selected a single population in each of the selection regimes and so we cannot fully account for the potential effect of genetic drift occurring within the populations, though future work using replicated selection populations could do this. It is notable that we achieved a rapid response to selection, detecting these effects after just 10 generations, but this is similar to the rapid response to selection for dauer larva formation that has been seen before [25], though the PDRP selection regime 1 was somewhat weaker in this regard. Other studies with *C. elegans* have selected for early reproduction, finding a strong response to this selection [26]. This is compatible with our results, where early reproduction was successfully selected for, though in this case in a pheromone-dependent manner. Other work selecting for early reproduction has shown now many sub-components of reproduction (such as production of hermaphrodite sperm, oogenesis and ovulation rate, and embryo retention times) are malleable traits which respond to selection [27].

*C. elegans* lives in ephemeral, boom-and-bust environments where larval arrest allows survival in the absence of food, and then rapid adult reproduction allows worms to exploit patches of food [17]. Different *C. elegans* strains live within these environments [28] and we suggest that pheromone-based signalling is used by worms to both cooperate and to compete in maximizing individuals’ fitness by affecting multiple life-history traits [17]. While these pheromone signals are publically broadcast into the environment we suggest that their meaning and correct interpretation is private among those interacting strains. An ecological approach is therefore now required to understand the role and adaptive value of pheromone signalling in *C. elegans* and nematodes more widely*.*

## Conclusion

The results presented here demonstrate that *C. elegans* pheromone can accelerate hermaphrodites’ reproductive rate, a phenomenon we call pheromone-dependent reproductive plasticity (PDRP). This is new role for this pheromone. The PDRP phenomenon is common to different *C. elegans* strains, though its magnitude differs among strains. We used a selection experiment to see how selecting for PDRP affected pheromone’s other role, inducing larval arrest, (and vice versa), showing that these two pheromone-dependent traits are not separate targets of selection. Collectively, these findings, with those of others, clearly shows that *C. elegans* pheromone affects multiple, important aspects of its life-history, which further suggests that to fully understand this pheromone signalling in *C. elegans* biology requires a more ecologically-based mode of study.

## Methods

### *C. elegans* strains, maintenance and assaying of reproduction

*C. elegans* strains MY1, N2, and PX174, and *E. coli* OP50, were obtained from the *Caenorhabditis* Genetics Center, and JU1409 and JU1410 from Marie-Anne Félix. Worms were maintained and assayed on NGM agar with an *E. coli* OP50 food source. We tested the effect of pheromone on worms’ reproduction by growing them individually on *E. coli*-seeded NGM agar that was supplemented with pheromone, or with water for controls, as [29]. For assays, 100 μL of pheromone (which is half that used in dauer formation assays as [17]) (or of water, for controls) was added to each 2 mL of agar in 35 mm diameter Petri dishes. Strains JU1401, MY1, N2 and PX174-derived pheromone is as [17]. *E. coli* OP50 was grown freshly in liquid LB media of which 100 μL was used to seed each assay plate. Worms were moved each day to a fresh plate, and the plate from which they had been removed was kept for 2 days at 19 °C to allow eggs to hatch into larvae, which were then counted.

In some assays we used a dead bacterial food source. To do this, bacteria (grown as above) were sedimented by centrifugation, resuspended as a ten times concentrated solution in water and 100 μL added to NGM-Kanamycin (final concentration of 50 μg/mL) agar plates; the bacterial slurry was allowed to dry and the plates then UV-exposed at a ~ 1000 mJ/cm^2^ dose [30].

To measure worms’ growth, synchronised, un-fed L1s were introduced to food on day 0, with 5 worms on each plate, and 12, 24, 36, 48, 60, 72, 96, 120, 168 h later worms were mounted on 0.1% (*w*/*v*) sodium azide-containing agar pads, photographed and worm length was measured using Image J. Vulval developmental stage after 36 h (above) was determined as [29].

In all assays, worms were synchronised by standard bleaching, and all assays performed at 19 °C.

### Statistical analyses of reproduction

ANOVA analyses of the data were performed in R using packages *lme4* [31, 32]. Post hoc Tukey comparisons were used to compare specific Strain and Treatment (presence or absence of pheromone) effects.

Generalised Linear Models and Generalised Linear Mixed-effects Models (GLMM) were fitted to the daily fecundity data, with GLMM only necessary to account for Block. The models had fixed effects of Day, Strain and Treatment, with Day treated as a categorical variable. Models were compared using Akaike’s Information Criterion [32]. To control for strains’ differences in total lifetime fecundity, fecundity was expressed as a daily proportion of their lifetime fecundity, and so in analyses of these data a binomial link function was used. Block was used as a random effect to account for the block design used. Differences between models were tested with log-likelihood ratio tests and where appropriate degrees of freedom (d.f.), χ^2^ value and *P* values are reported. Worm growth rate assays were analysed by comparing worm lengths at each time point using Welch’s t-test, producing non-integer degrees of freedom. Because destructive sampling was used, each data point is independent.

### Selection experiment

The aim of this selection experiment was to ask how separate selection on each of the two pheromone-dependent phenotypically plastic traits (PDRP and dauer larva formation), would affect (or not) (i) the target trait, and (ii) the non-target trait. We used the genetically diverse *C. elegans* G140a population [33] as the base population for selection. Populations were selected for 10 generations in one of three selection regimes.

Selection regime 1, PDRP. This selection regime selected worms for PDRP. The rationale for this was to select for pheromone-dependent plasticity of reproduction and given that pheromone hastens when worms maximise their reproduction, this selection alternated between selecting for fast reproduction in the presence of pheromone, and slow reproduction in the absence of pheromone. Specifically, to do this, in generation 1 worms were grown for 3 days in the presence of 20 μL pheromone per 2 mL of agar before selection for eggs to found the next generation; in generation 2 worms were maintained or 5 days in the absence of pheromone before selection for eggs to found the next generation. This alternating, 3 days with pheromone, 5 days without pheromone was continued for 10 generations (Fig. 4a).

Selection regime 2, Dauer. This selection regime selected worms for dauer larva formation, and here the rationale was to select worms that were sensitive to pheromone with respect to the formation of dauer larvae. In addition, to be consistent with selection regime 1, and the control (below), alternating generations were selected for slow adult reproduction. Specifically, to do this, in generation 1 worms were exposed to a dauer larva-inducing environment (the presence of 40 μL pheromone per 2 mL of agar, and limiting food which was a 2% *w*/*v* dilution of an overnight culture of *E. coli* OP50, as [17], and a temperature of 25 °C), for 3 days after which dauer larvae were selected as [25]. These dauer larvae were then allowed to recover by being supplied with abundant food in the absence of pheromone, before selection for eggs to found the next generation. In generation 2, worms were maintained for 5 days in the absence of pheromone before selection for eggs to found the next generation. This alternating selection was continued for 10 generations (Fig. 4a).

Selection regime 3, Control. This selection regime was the control for selection regimes 1 and 2. The rationale for this regime was to select for fast and then slow adult reproduction in the absence of pheromone. To do this, in the absence of pheromone, in generation 1, worms were grown for 3 days before selection for eggs to found the next generation; in generation 2 worms were grown for 5 days before selection for eggs to found the next generation. This alternating 3 then 5 day periods of growth before selection was continued for 10 generations (Fig. 4a).

Throughout, these populations were maintained at a size of 10,000 worms, across ten 9 cm diameter Petri dishes, each containing 1000 worms, where possible. Pheromone was produced from strain N2, as [17], and the batch used in this selection experiment is different than the batch used in the PDRP analyses. The entire experiment was conducted at 19 °C, except where indicated in selection regime 2 (above). In all selection regimes, at the point of selection all worms were harvested from plates and viable eggs obtained as [25], which were then used to initiate the next population for selection.

After the 10 generations of selection, 5 individual worms were randomly selected from each selected population and each inbred for 10 generations, to make near-isogenic lines. These 15 lines were then phenotyped for their PDRP as described above, except that 20 μL of pheromone (or water) was used for each 2 mL of agar with triplicate assays for each of the 15 lines. Analogously, the 15 lines’ dauer larva arrest phenotypes were determined as [17] (using 40 μL pheromone per 2 μL agar, with 20 μL of a 2% *w/v* dilution of the bacterial food source, as above) with three assay plates used for each of the 15 lines.

### Statistical analysis of the selection experiment

These analyses sought to test how the different selection regimes (Selection Regime) affected the selected worms’ dauer larva formation phenotype and their reproductive phenotype. For the dauer larvae formation data, the proportion of larvae that formed dauer larvae were modelled using GLMs with a binomial link function. For the reproductive phenotypes, the worms’ fecundity on each of its four days of reproduction was expressed as a proportion of a worm’s lifetime fecundity and this modelled for each day separately using GLMs with a binomial link function. For both traits, differences between models were tested using log-likelihood ratio tests, with Tukey post hoc analysis used to compare individual selection regimes.

## Abbreviations

GLMM: Generalised Linear Mixed-effects Models
L: Larval stage
PDRP: Pheromone-Dependent Reproductive Plasticity
SD: Standard Deviation
